# Modulating heart rate oscillation affects plasma amyloid beta and tau levels in younger and older adults

**DOI:** 10.1038/s41598-023-30167-0

**Published:** 2023-03-09

**Authors:** Jungwon Min, Jeremy Rouanet, Alessandra Cadete Martini, Kaoru Nashiro, Hyun Joo Yoo, Shai Porat, Christine Cho, Junxiang Wan, Steve W. Cole, Elizabeth Head, Daniel A. Nation, Julian F. Thayer, Mara Mather

**Affiliations:** 1 University of Southern California, Los Angeles, CA USA; 2 University of California, Irvine, Irvine, CA USA; 3 University of California, Los Angeles, Los Angeles, CA USA

**Keywords:** Psychology, Human behaviour, Biomarkers, Predictive markers, Alzheimer's disease, Ageing

## Abstract

Slow paced breathing via heart rate variability (HRV) biofeedback stimulates vagus-nerve pathways that counter noradrenergic stress and arousal pathways that can influence production and clearance of Alzheimer's disease (AD)-related proteins. Thus, we examined whether HRV biofeedback intervention affects plasma Αβ40, Αβ42, total tau (tTau), and phosphorylated tau-181 (pTau-181) levels. We randomized healthy adults (N = 108) to use slow-paced breathing with HRV biofeedback to increase heart rate oscillations (Osc+) or to use personalized strategies with HRV biofeedback to decrease heart rate oscillations (Osc−). They practiced 20–40 min daily. Four weeks of practicing the Osc+ and Osc− conditions produced large effect size differences in change in plasma Aβ40 and Aβ42 levels. The Osc+ condition decreased plasma Αβ while the Osc− condition increased Αβ. Decreases in Αβ were associated with decreases in gene transcription indicators of β-adrenergic signaling, linking effects to the noradrenergic system. There were also opposing effects of the Osc+ and Osc− interventions on tTau for younger adults and pTau-181 for older adults. These results provide novel data supporting a causal role of autonomic activity in modulating plasma AD-related biomarkers.

Trial registration: NCT03458910 (ClinicalTrials.gov); first posted on 03/08/2018.

## Introduction

Alzheimer’s disease (AD) incidence rates increase exponentially with age^1^. Why does aging increase AD risk so much? One potentially critical factor has been given little attention. During aging, the balance between the sympathetic and parasympathetic branches of the autonomic nervous system shifts^2,3^. As people get older, parasympathetic activity declines, as indicated by decreases in heart rate variability (HRV)^2^. At the same time, sympathetic (or noradrenergic) activity increases, as indicated by increases in sympathetic nerve activity and circulating noradrenaline levels^4^. Age-related increases in noradrenergic activity and decreases in parasympathetic activity are associated with AD-related conditions including sleep disorders, diabetes, and heart disease^5^.

Age-related increases in noradrenergic activity along with decreases in parasympathetic activity might influence levels of amyloid-β (Aβ) peptides in the brain and body^6^. Generally, increasing neuronal or cellular activity stimulates release of Aβ^7^. Rodent AD models indicate that noradrenergic agonists/antagonists affect Aβ accumulation and amyloid plaque formation^8,9^ and suggest that stressful situations tend to stimulate Aβ peptide release into the interstitial fluid^10^. While these findings suggest that countering noradrenergic activity could help decrease Aβ release in the brain, predictions involving tau proteins are not straightforward. Similar to Aβ, neuronal activity increases tau release^11–13^ and repeated stress induces tau phosphorylation^14^. However, research indicates that anesthetics that lower noradrenergic activity induce tau phosphorylation^15,16^, and dexmedetomidine, an ⍺2 adrenergic receptor agonist that produces a sedative state, also increases tau phosphorylation^17^. In addition, animal studies suggest that arousal states affect brain waste clearance by modulating effectiveness of glymphatic pathways which transport cerebrospinal fluid (CSF) and flush interstitial waste from the brain to veins^18,19^. Glymphatic transport was increased when inducing anesthesia with dexmedetomidine, suppressing noradrenaline release^20^ and when administering adrenergic antagonists^19^. Furthermore, stimulating the vagus nerve, which provides parasympathetic innervation, increased CSF penetrance in the brain^21^. Similar dynamics may exist in human brains^22,23^. Certainly, sleep affects Aβ levels. One night of sleep disruption increased Aβ concentrations in the CSF^24,25^, and older adults with lower slow-wave activity during sleep had higher Aβ and tau accumulation measured by positron emission tomography (PET) scans^26^. However, the effects of sleep or sleep deprivation may be more related to production than clearance. For instance, measuring Aβ stable isotope labeling kinetics suggest that sleep deprivation increases Aβ production^27^. Together, these studies suggest that enhancing parasympathetic activity either via improving sleep or directly stimulating the vagus nerve has the potential to reduce Aβ and tau levels.

The vagus nerve can be non-invasively stimulated by breathing around the baroreflex frequency (0.1 Hz or 10 s/breath)^28^. The 10 s-paced breathing can stimulate brain mechanisms that help control blood pressure and heart beats and boost the amplitude of cardiovascular oscillations^29^. We hypothesized that, by attenuating noradrenergic activity and enhancing parasympathetic activity, the amplified oscillations could reduce Aβ release and facilitate clearing the aggregation-prone Aβ42 and pTau-181 from the brain to the periphery. To test these possibilities, we added measures of plasma Aβ and tau to a clinical trial involving daily sessions of HRV biofeedback (ClinicalTrials.gov NCT03458910; primary study outcomes were focused on effects on emotion-related brain networks)^30^. As outcomes that would reflect changes in cellular release of Aβ, we examined plasma Aβ40 and Aβ42 levels. We also included plasma total tau (tTau) and phosphorylated tau (pTau-181) to measure effects on tau proteins. As outcomes that would reflect changes in clearance from the brain to blood, we examined two plasma ratios: Aβ42 to Aβ40 and pTau-181 to tTau. We selected these as clearance-related outcomes as these ratios each involve one plasma biomarker that is more likely to be brain-derived (Aβ42, pTau-181) and one that relates more to peripheral production or release (Aβ40, tTau). Compared with Aβ40, Aβ42 is relatively more prevalent in the brain than in the periphery^31^. Platelets are the major source of peripheral Aβ^7,32^ and produce predominantly Aβ40 over Aβ42^7,31^. Plasma Aβ42 is more likely than plasma Aβ40 to reflect brain-derived Aβ, as reflected in the stronger correlation between plasma and CSF Aβ42 than between plasma and CSF Aβ40^33,34^. Plasma pTau-181 and tTau show a similar brain vs. periphery dichotomy. Once diagnostic groups are controlled for, plasma tTau and CSF tTau do not significantly correlate with each other, and are correlated with different aspects of brain atrophy and cognition^33,35–37^. In contrast, plasma pTau-181 levels correlate with CSF pTau-181 levels^38,39^, suggesting plasma pTau-181 is more likely than plasma tTau to originate in the brain.

To test whether the HRV biofeedback intervention could affect Aβ and tau dynamics, we conducted assays of plasma samples from 108 healthy adults (54 younger and 54 older adults) who were randomized into one of two groups with opposing goals: reducing or increasing the amplitude of heart rate oscillations (Osc− vs. Osc+ condition). In addition to testing effects across age groups, we conducted follow-up analyses separately for younger and older adults. To our knowledge, no prior studies have compared the effects of any interventions across younger vs. older adults on AD biomarkers. However, analyzing the data separately for the two age groups could provide important insights regarding which effects are general across adulthood and which are specific to one age group. In addition, we ran two exploratory analyses. First, to test whether intervention effects on plasma biomarkers were related to changes in noradrenergic activity, in the younger subgroup of the participants (N = 54), we conducted gene expression analyses of circulating blood cells to assess longer-term tonic levels of noradrenergic activity. We used cAMP-responsive element binding protein (CREB)-regulated gene expression as a slow-moving index of β-adrenergic signaling that reflects adrenergic-related transcription activity^40^. Second, we examined the possible association between change in Aβ42/40 ratios and negative emotion as studies indicated that plasma Aβ42/40 ratios are correlated with later-life major depression^41,42^.

## Results

### Baseline characteristics of participants

The male and female ratio was similar across age groups and conditions (Table 1). At baseline, in neither age cohort did the Osc+ and Osc− groups differ significantly in plasma Aβ, plasma tau, age, body mass index, waist hip ratio, blood pressure, sleep, nor heart rate variability (Table 2). In addition, we compared males to females for the same baseline measures of Table 2 and reported the results in Supplementary Table. There was no significant baseline difference in CREB, bootstrap *Z* = 1.31, *p* = 0.19 (available only for 54 younger adults).

**Tab﻿le 1:** Age group and sex for Osc+ vs. Osc−.

	Osc+	Osc−	Total
Younger adults			
Male	11	7	18
Female	19	17	36
Total	30	24	54
Older adults			
Male	7	8	15
Female	20	19	39
Total	27	27	54

**Table 2:** Comparison of baseline characteristics between Osc+ and Osc−.

	Osc+		Osc−		Test statistics		
	M	SE	M	SE	*t*	*df*	*p*
Younger adults							
Age (years)	22.60	0.47	22.70	0.73	−0.12	40.3	0.91
Plasma Ab42 (pg/ml)	9.76	0.43	9.26	0.47	0.79	52	0.43
Plasma Ab40 (pg/ml)	166.95	7.58	151.75	4.95	1.68	48.0	0.10
Plasma Ab42/Ab40 ratio	0.06	0.002	0.06	0.003	−0.62	52	0.54
Plasma tTau (pg/ml)	2.13	0.14	2.11	0.16	0.12	52	0.90
Plasma pTau-181 (pg/ml)	1.57	0.12	1.57	0.14	0.02	52	0.98
Plasma pTau-181/tTau ratio	0.78	0.06	0.77	0.06	0.12	52	0.90
Body mass index (kg/m^2^)	23.44	0.70	23.43	0.97	0.003	51	0.997
Waist hip ratio	0.80	0.01	0.79	0.01	0.88	52	0.38
Systolic blood pressure (mmHg)	113.58	2.02	110.90	2.09	0.92	52	0.36
Diastolic blood pressure (mmHg)	69.22	1.45	70.98	1.35	−0.87	52	0.39
Mean heart rate (beat/min)	71.78	2.06	73.29	1.90	−0.53	52	0.60
RMSSD (ms)	67.64	5.93	57.16	3.92	1.48	48.3	0.15
Hours of sleep (h)	6.41	0.17	6.08	0.20	1.26	45	0.21
Hours of REM sleep (h)	1.43	0.09	1.39	0.11	0.29	45	0.77
Hours of deep sleep (h)	1.13	0.11	1.16	0.09	−0.22	45	0.82
Heart rate during deep sleep (beat/min)	59.82	1.15	60.86	1.55	−0.54	45	0.59
RMSSD during deep sleep (ms)	82.64	6.89	76.1	5.86	0.72	45	0.48
Cortisol at awakening (µg/dl)	0.29	0.03	0.25	0.04	0.73	49	0.47
Cortisol awakening response (µg/dl)	0.19	0.05	0.25	0.06	−0.87	47	0.39
Blood collection time (h)	14.43	0.13	14.30	0.11	0.73	52	0.47
Older adults							
Age (years)	66.19	1.56	65.65	1.14	0.28	52	0.78
Plasma Ab42 (pg/ml)	10.78	0.58	10.94	0.62	−0.19	52	0.85
Plasma Ab40 (pg/ml)	198.02	9.15	211.76	23.47	−0.54	52	0.59
Plasma Ab42/Ab40 ratio	0.05	0.001	0.05	0.002	−0.330	52	0.74
Plasma tTau (pg/ml)	1.91	0.12	2.17	0.22	−1.04	40.99	0.30
Plasma pTau-181 (pg/ml)	2.55	0.22	2.64	0.31	−0.25	52	0.81
Plasma pTau-181/tTau ratio	1.42	0.15	1.39	0.14	0.14	52	0.89
Body mass index (kg/m^2^)	26.40	0.99	26.54	1.36	−0.09	52	0.93
Waist hip ratio	0.87	0.04	0.86	0.02	0.23	52	0.82
Systolic blood pressure (mmHg)	126.63	3.13	129.04	2.69	−0.58	52	0.56
Diastolic blood pressure (mmHg)	77.26	1.80	78.44	1.65	−0.49	52	0.63
Mean heart rate (beat/min)	67.93	1.91	71.88	2.05	−1.38	47	0.17
RMSSD (ms)	35.71	4.10	32.86	2.90	0.58	47	0.56
Hours of sleep (h)	6.04	0.22	5.63	0.28	1.14	43	0.26
Hours of REM sleep (h)	1.52	0.12	1.45	0.10	0.48	43	0.63
Hours of deep sleep (h)	1.18	0.06	1.12	0.08	0.64	43	0.52
Heart rate during deep sleep (beat/min)	59.41	1.65	59.05	7.02	−1.85	43	0.07
RMSSD during deep sleep (ms)	59.05	7.02	44.52	4.61	1.71	43	0.09
Blood collection time (h)	12.34	0.20	11.82	0.24	1.65	52	0.10

We then tested age differences in the six biomarkers (Aβ42, Aβ40, tTau, pTau-181, Aβ42/Aβ40 ratio and pTau-181/tTau ratio) at baseline. We adjusted *p* values to control for false discovery rate^43,44^. Older adults compared with younger adults showed higher baseline levels of Aβ42, *t*(106) = 2.51, adjusted *p* = 0.01, Aβ40, *t*(106) = 3.33, adjusted *p* = 0.002, pTau-181, *t*(76.4) = 4.95, adjusted *p* = 0.00004, and pTau/tTau ratios, *t*(71.0) = 5.65, adjusted *p* = 0.000006, but lower baseline Aβ42/Aβ40 ratios, *t*(85.0) = −3.02, adjusted *p* = 0.005, and no significant differences in tTau, *t*(106) = −0.52, adjusted *p* = 0.33.

### Intervention effects on Aβ42 and Aβ40

There were significant time-point by condition interactions for Aβ42, *F*(1, 106) = 16.62, adjusted *p* = 0.0005, *η*_p_^2^ = 0.14, and for Aβ40, *F*(1, 106) = 14.01, adjusted *p* = 0.001, *η*_p_^2^ = 0.12. Simple main effects showed that Osc+ decreased Aβ42 (*p* = 0.05) and Aβ40 levels (*p* = 0.03) from pre- to post-intervention, while Osc− increased Aβ42 (*p* < 0.001) and Aβ40 levels (*p* = 0.003). With conditions collapsed, there were no main time-point effects for either Aβ42 (*p* = 0.17) or Aβ40 (*p* = 0.50). There was no interaction for the Aβ42/Aβ40 ratio, *F*(1, 106) = 0.55, adjusted *p* = 0.26, *η*_p_^2^ = 0.01, with no main time-point effect (*p* = 0.89). The significant results were maintained after removing outliers (Supplementary Information).

**Figure 1:**
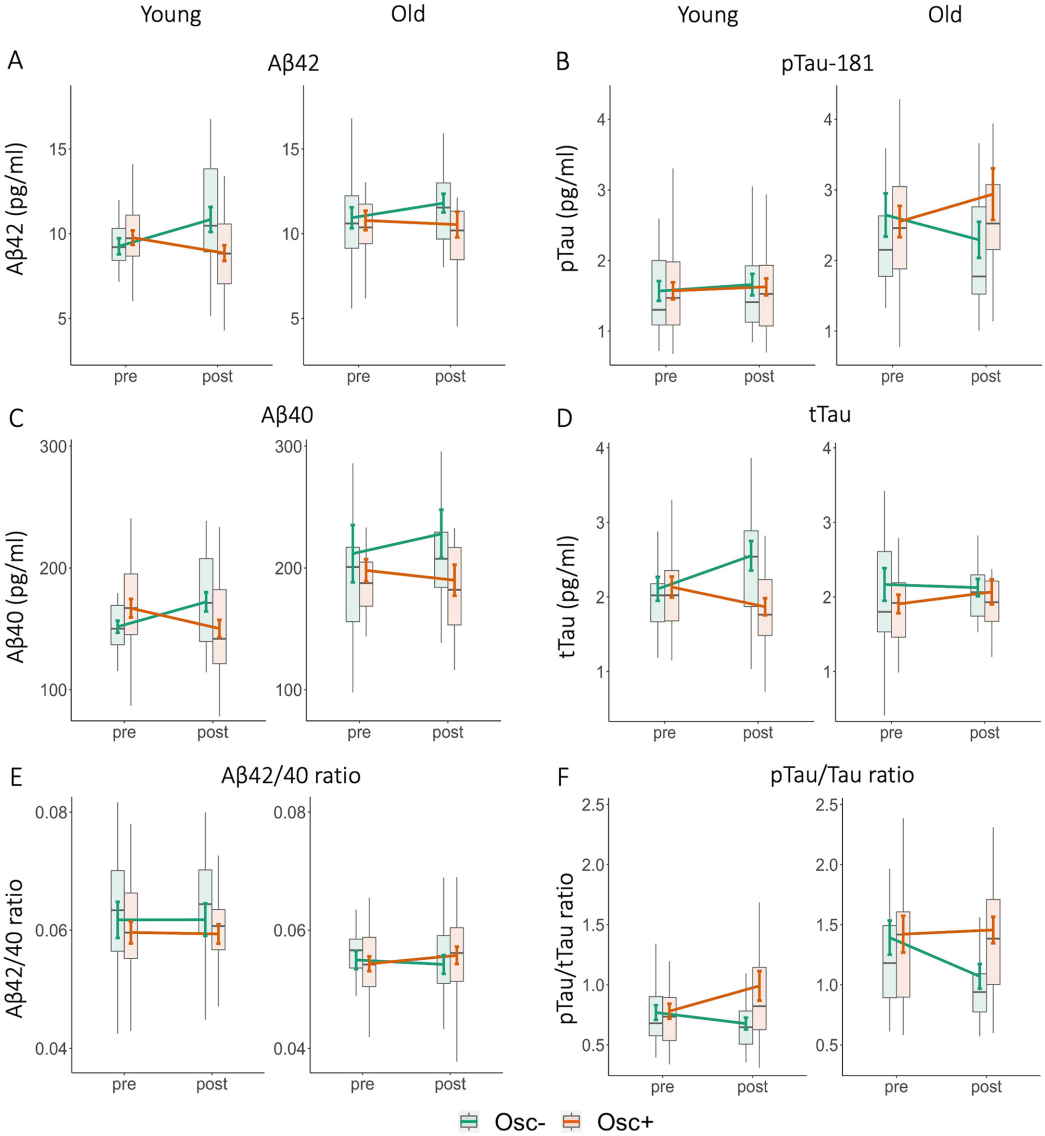
Intervention effect on Aβ and tau levels. Orange represents Osc+ and green represents Osc−. The upper and lower box boundaries indicate the 75th and 25th percentiles respectively. The gray horizontal bar inside each box shows a median value for the box, and the colored vertical line describes the mean and standard error. The outliers are included for the summary statistics but are not shown in the figure.

In follow-up tests where we split the data (N = 108) into the younger (N = 54) and older (N = 54) age groups (Fig. 1), the time-point by condition interactions on Aβ levels remained significant for younger adults, *F*(1, 52) = 12.30, adjusted *p* = 0.002, *η*_p_^2^ = 0.19 for Aβ42 and *F*(1, 52) = 6.64, adjusted *p* = 0.01, *η*_p_^2^ = 0.11 for Aβ40 as well as for older adults, *F*(1, 52) = 4.51, adjusted *p* = 0.04, *η*_p_^2^ = 0.08 for Aβ42 and *F*(1, 52) = 7.37, adjusted *p* = 0.01, *η*_p_^2^ = 0.12 for Aβ40. For the Aβ42/Aβ40 ratio, older adults showed a trend towards an interaction, *F*(1, 52) = 3.35, adjusted *p* = 0.05, *η*_p_^2^ = 0.06, whereas younger adults showed no interaction effect, *F*(1, 52) = 0.02, adjusted *p* = 0.45, *η*_p_^2^ < 0.001.

### Intervention effects on tTau and pTau-181

Across all participants, there were no significant time-point by condition interactions for either tTau, *F*(1, 106) = 2.01, adjusted *p* = 0.10, *η*_p_^2^ = 0.02 or pTau-181, *F*(1, 106) = 3.28, adjusted *p* = 0.05, *η*_p_^2^ = 0.03. However, the pTau/tTau ratio showed a significant interaction, *F*(1, 106) = 7.41, adjusted *p* = 0.01, *η*_p_^2^ = 0.07. Simple main effects showed that Osc− decreased the pTau/tTau ratio (*p* = 0.02) from pre- to post-intervention, while Osc+ increased it but not significantly (*p* = 0.14). With conditions collapsed, there were no main time-point effects (*p* = 0.49).

The follow-up analyses for younger and older adults (Fig. 1) revealed a significant time-point by condition interaction in tTau for younger adults, *F*(1, 52) = 8.63, adjusted *p* = 0.007, *η*_p_^2^ = 0.14, but not for older adults, *F*(1, 52) = 0.69, adjusted *p* = 0.24, *η*_p_^2^ = 0.01. For pTau-181, younger adults showed no interaction, *F*(1, 52) = 0.02, adjusted* p* = 0.45, *η*_p_^2^ < 0.001, while older adults showed a significant interaction, *F*(1, 52) = 5.55, adjusted* p* = 0.02, *η*_p_^2^ = 0.10. For the pTau/tTau ratio, neither younger nor older adults showed significant interactions, *F*(1, 52) = 3.56, adjusted *p* = 0.05, *η*_p_^2^ = 0.06 for younger adults and *F*(1, 52) = 3.46, adjusted *p* = 0.05, *η*_p_^2^ = 0.06 for older adults.

Based on our hypothesis that changes in the Aβ42/Aβ40 ratio and the pTau/Tau ratio could both be affected by changes in brain clearance, we expected some correlation in change among these two ratio scores. Changes in the pTau/Tau ratio correlated in the positive direction with changes in the Aβ42/Aβ40 ratio when controlled for their baseline values across all participants, *r*(104) = 0.20, adjusted *p* = 0.04. However, the correlation between ratio scores was not significant independently for younger adults, *r*(50) = 0.20, adjusted *p* = 0.10, nor for older adults, *r*(50) = 0.20, adjusted *p* = 0.10.

### Relationship between changes in plasma Aβ levels and noradrenergic-related gene transcription activity

Among the younger subset tested (N = 54), there was a significant time-point by condition interaction in CREB transcription factor activity, bootstrap *Z* = −3.30, adjusted *p* = 0.002. While Osc+ showed no significant changes (*Z* = −0.45, *p* = 0.65), Osc− increased CREB activity after intervention (*Z* = 2.70, *p* = 0.008). When we examined the correlations between changes in CREB activity and changes in the six biomarkers, we found that individual decreases in plasma Aβ40 from pre- to post-intervention were associated with decreases in activity of the CREB transcription factor that mediates sympathetic nervous system-induced β-adrenergic signaling (0.75-fold prevalence of CREB response elements in Aβ40 down-regulated vs. up-regulated genes; 0.418 log2 ratio units ± 0.120 standard error; *Z* = 2.98, adjusted *p* = 0.002). Decreases in CREB activity were not significantly associated with individual decreases in plasma Aβ42 from pre- to post-intervention (0.87-fold; 0.207 ± 0.114; *Z* = 1.82, adjusted *p* = 0.05) and thus showed an inverse association with the Aβ42/Aβ40 ratio (1.32-fold; −0.392 ± 0.116; *Z* = −3.39, adjusted *p* = 0.002). Increases in pTau-181 were associated with decreased CREB activity (1.35-fold; −0.427 ± 0.120; *Z* = −3.56, adjusted *p* = 0.002). Neither tTau (1.18-fold; −0.237 ± 0.136; *Z* = −1.75, adjusted *p* = 0.06), nor pTau-181/tTau ratios (1.06-fold; −0.083 ± 0.101; *Z* = −0.81, adjusted *p* = 0.24) showed a significant association with CREB activity.

### Individual differences in plasma Aβ42/40 and negative affect

There was no intervention effect for negative affect, *F*(1, 50) = 0.32, *p* = 0.58, *η*_p_^2^ = 0.01 for younger adults and *F*(1, 45) = 0.73, *p* = 0.40, *η*_p_^2^ = 0.02 for older adults. However, pre- to post-intervention increases in Aβ42/40 ratio were associated with decreases in negative affect, *r*(43) = −0.41, adjusted *p* = 0.007 for older adults, but not for younger adults, *r*(48) = −0.02, adjusted *p* = 0.45.

### Intervention effects on other physiological variables

Other than plasma Aβ and tau changes, we did not see significant intervention-induced changes in any other physiological measures including BMI, blood pressure, RMSSD (root mean square of successive differences) at rest, total sleep time, REM (rapid eye movement) sleep, deep sleep, and RMSSD during deep sleep (all *p*’s > 0.05, Table 3).

**Table 3:** Comparison of pre- and post-intervention changes (post–pre) between Osc+ and Osc−.

	Osc+		Osc−		Test statistics		
	M	SE	M	SE	*T*	*df*	*p*
Younger adults							
Body mass index (kg/m^2^)	0.01	0.28	0.27	0.32	−0.610	50	0.55
Waist hip ratio	0.001	0.006	0.005	0.008	−0.40	52	0.69
Systolic blood pressure (mmHg)	−3.02	2.14	0.48	1.73	−1.23	52	0.23
Diastolic blood pressure (mmHg)	0.18	1.56	−1.00	1.18	0.58	52	0.56
Mean heart rate (beat/min)	−4.86	1.76	−9.03	2.40	1.17	52	0.25
RMSSD (ms)	−5.09	5.56	6.95	11.52	−1.00	52	0.32
Hours of sleep (h)	−0.12	0.18	0.12	0.12	−1.13	39.7	0.26
Hours of REM sleep (h)	0.03	0.07	0.06	0.07	−0.27	45	0.79
Hours of deep sleep (h)	0.03	0.10	−0.10	0.05	1.11	34.0	0.27
Heart rate during deep sleep (beat/min)	0.34	0.44	2.28	2.02	0.29	45	0.78
RMSSD during deep sleep (ms)	2.28	2.02	−1.45	2.44	1.18	45	0.24
Cortisol at awakening (µg/dl)	−0.02	0.05	0.02	0.04	−0.47	47	0.64
Cortisol awakening response (µg/dl)	0.03	0.07	−0.06	0.07	0.97	43	0.34
Blood collection time (h)	−0.24	−0.08	0.06	0.10	−1.37	52	0.18
Older adults							
Body mass index (kg/m^2^)	0.04	0.12	−0.49	0.41	1.23	47	0.23
Waist hip ratio	0.025	0.044	0.004	0.008	0.50	49	0.62
Systolic blood pressure (mmHg)	−0.23	3.36	−0.96	3.22	0.16	48	0.88
Diastolic blood pressure (mmHg)	−0.60	1.62	−1.21	1.27	0.30	48	0.77
Mean heart rate (beat/min)	1.36	1.08	1.84	1.23	−0.29	47	0.77
RMSSD (ms)	14.84	9.41	6.46	4.27	0.86	47	0.39
Hours of sleep (h)	−0.41	0.21	−0.28	0.22	−0.41	43	0.68
Hours of REM sleep (h)	−0.10	0.07	−0.10	0.08	−0.05	43	0.96
Hours of deep sleep (h)	−0.07	0.05	−0.05	0.05	−0.30	43	0.77
Heart rate during deep sleep (beat/min)	−0.84	0.53	−0.98	0.56	0.18	43	0.86
RMSSD during deep sleep (ms)	−1.33	2.43	0.04	2.01	−0.43	43	0.67
Blood collection time (h)	−0.04	0.11	−0.11	0.09	0.55	52	0.58

## Discussion

The current study investigated whether modulating heart rate oscillations via HRV biofeedback would affect plasma Aβ and tau levels. The study showed that daily practice of inducing high-amplitude heart rate oscillations (Osc+) reduced plasma Aβ42 and Aβ40 levels, while daily practice of reducing heart rate oscillations (Osc−) raised plasma Aβ42 and Aβ40 levels. These interaction effects of time and condition were significant both for younger and older adults. Plasma tTau also showed reductions in the Osc+ condition and increases in the Osc− condition among younger adults but showed no significant effects among older adults. Gene expression analysis in younger adult samples revealed that the magnitude of decreases in plasma Aβ40 was associated with the downregulation of CREB transcription factor activity. Even though the intervention did not affect pTau-181 levels in younger adults, increases in pTau-181 were associated with decreases in CREB activity, consistent with prior findings that anesthesia, a state of low noradrenergic activity, induced tau phosphorylation^15,16^. CREB-regulated gene expression in circulating blood cells provides a slow-moving time-integrated index of β-adrenergic signaling^40^ that reflects noradrenergic activity during the intervention period, thus these findings suggest that changes in these plasma biomarkers were associated with changes in noradrenergic activity.

As far as we can tell from the published literature and ClinicalTrials.gov searches, this study provides the first evidence of a behavioral intervention that reduces Aβ levels (measured with plasma, CSF or PET) compared to a randomized control group. For instance, at least to date, exercise interventions have not decreased Aβ levels (nor improved the plasma Aβ42/Aβ40 ratio)^45–48^. In healthy adults, higher plasma Aβ40 and Aβ42 levels predict higher risk of an AD diagnosis^49,50,^and high plasma Aβ40 levels appear to promote vascular aging^51^ and are associated with increased risk of mortality^52^. Slow-paced breathing via HRV biofeedback may be a low-cost and low-risk way to reduce plasma Aβ levels. Effects of the interventions on plasma Aβ levels were seen not only among older adults but also among younger adults. Thus, regularly practicing slow paced breathing via HRV biofeedback may help keep plasma Aβ levels low throughout adulthood.

In addition to testing overall levels of plasma Aβ and tau, we also examined whether the interventions affected plasma Aβ42/Aβ40 and pTau-181/tTau ratios. We were interested in these two ratio scores as proxy measures of brain clearance, given the greater likelihood of plasma Aβ42 and pTau-181 to be brain-derived compared with Aβ40 and tTau, respectively^33,34,38,39^. The Aβ42/Aβ40 ratio showed a trend towards an interaction effect in older adults only, for whom the ratio increased for Osc+ relative to Osc−. For older adults, increased Aβ42/Aβ40 ratios were significantly associated with decreased negative affect, consistent with the literature linking plasma Aβ42/Aβ40 ratios and depression^41,42^. The pTau-181/tTau ratio showed significant differences in change between the two intervention groups across age groups, with effects not significant in either age group alone. The condition differences in the ratio score were driven by changes in tTau for younger adults and by changes in pTau-181 for older adults (Fig. 1). Although suggestive, our findings regarding the potentially clearance-related plasma Aβ42/Aβ40 and pTau-181/tTau ratios do not provide clear support that the interventions affected brain clearance as the Aβ42/Aβ40 changes did not quite achieve significance and there are other potential dynamics that could affect pTau-181/tTau ratios other than changes in brain clearance. For instance, greater plasma pTau-181 levels are typically viewed as reflecting worse brain pathology, as plasma pTau-181 correlates positively with PET measures of amyloid and tau burden^53^. Also, as already discussed, previous findings indicate noradrenergic influences on tau phosphorylation^15–17^. Thus, the pTau-181/tTau ratio measure is ambiguous. While our findings indicate that daily practice sessions aimed at modulating heart rate oscillation differently affect plasma pTau-181 and tTau, the mechanisms are unclear. To address the possibility of clearance-related effects, future studies should include more direct brain measures of amyloid/tau burden or clearance dynamics.

Taken together, our results provide novel evidence that manipulating autonomic activity affects plasma AD biomarkers. There are at least three plausible (and potentially synergistic) routes of influence that should be tested in future studies: (1) production; (2) peripheral clearance; and (3) brain clearance.

First, high-amplitude heart rate oscillations induced by slow-paced breathing could lessen Aβ production by reducing noradrenergic activity^6^. In general, experiencing stress and adversity has been associated with higher risk of AD in humans and rodents^10^, and the tonic pattern of activity seen in the locus coeruleus (the source of most of the brain’s noradrenaline) under stress accelerates spread of AD pathology compared with a non-stress phasic pattern of activity^54^. Our findings of an association between changes in the plasma Aβ and changes in the CREB transcription factor activity are consistent with the possibility that noradrenergic activity plays a role in these changes. An interesting question is whether reduced noradrenergic activity during the intervention practice sessions affects Aβ production or whether changes in tonic resting-state noradrenergic activity mediate the outcomes. In the current study, HRV biofeedback interventions did not change resting-state physiological states including RMSSD and heart rate (Table 3) and, in exploratory post-hoc analyses, changes in RMSSD and heart rate did not significantly correlate with changes in plasma Aβ or tTau levels (Supplementary Information). However, decreases in plasma Aβ and tTau levels correlated with the intensity of heart rate oscillations during practice (Supplementary Information). Thus, initial findings suggest that the changes in overall levels of plasma Aβ and tTau depended more on the strong differences in oscillatory dynamics elicited during practice sessions than on changes in resting-state physiology.

Second, the heart rate oscillations could modulate peripheral clearance functions involving the kidney. The Osc+ intervention was designed to maximize heart rate oscillations by aligning breathing rate with the baroreflex frequency. Prior studies indicated that stimulating the baroreflex suppresses chronic sympathetic nerve activity in the kidney, which then facilitates its excretion of bodily waste including Aβ and tau^55,56^. Importantly, even if the intervention effects were entirely driven by peripheral mechanisms, reducing Aβ and tau in the periphery is likely to have downstream benefits for clearing Aβ and tau derived in the brain^57^.

Third, slow-paced breathing may stimulate the clearance of Aβ and tau from the brain. The majority of Aβ isoforms are removed via transport across the blood brain barrier and via the glymphatic system using the interstitial fluid bulk flow^58^. Flow of cerebrospinal fluid through the glymphatic system is enhanced by decreased noradrenergic activity^19^ and by breathing- and/or heartbeat-induced pulsatile waves of blood flow^59^. These clearance systems decline with aging and AD-related pathology^60,61^. As discussed above, our findings were mixed regarding the effects of the interventions on potential plasma biomarker signals of change in clearance and future work should include more direct measures of clearance.

This study also had some limitations that should be addressed in future studies. One key limitation is that plasma Aβ and tau levels are indirect measures of brain Aβ and tau levels^62^. Future studies are needed to see if these manipulations involving heart rate oscillations impact Aβ and tau brain levels and their eventual aggregation. Next, plasma Aβ and tau levels can vary depending on multiple factors including sleep and circadian rhythms^63^. We minimized the impact of this potential variability by collecting blood at similar times for each participant at pre- and post-intervention and measuring sleep hours using an actigraph device. There were no significant pre-post intervention differences in blood collection times or sleep hours (Table 3). Finally, we note that the effects were driven by opposing effects of the Osc+ and Osc− interventions. Although these intervention effects on plasma biomarkers likely dissipate after the intervention no longer being practiced, to avoid the increased plasma Aβ levels found in the active control condition (Osc−), future longer-term studies may consider employing a neutral control condition.

In summary, we found that HRV biofeedback interventions modulated plasma Aβ and tau levels in younger and/or older adults, and that, at least among the younger adults, changes in plasma Aβ and tau levels were associated with changes in noradrenergic activity. There are multiple plausible pathways through which high-amplitude heart rate oscillations could affect plasma Aβ and tau; indeed, it is possible that the differences between the two conditions seen here are due to multiple synergistic pathways that simultaneously affect Aβ and tau production and clearance.

## Methods

### Study design and participants

The current study was part of a larger heart rate variability biofeedback intervention study (ClinicalTrials.gov Identifier: NCT03458910)^30^. Healthy participants without serious medical conditions participated in the seven-week study after providing informed consent approved by University of Southern California’s Institutional Review Board. The current report focuses on the 54 younger and 54 older adults who had blood samples available at both pre and post intervention. Recruiters, blind to condition assignment, allocated participants in waves of around 20 to small groups of 3–6 people such that each group could visit the lab on a common weekly schedule. Each group was randomly assigned to either the Osc+ or Osc− condition. Both conditions involved 20–40 min of daily home practice of HRV biofeedback with opposite goals. Osc+ participants were instructed to maximize their heart rate oscillations using slow paced breathing, while Osc− participants were instructed to minimize their heart rate oscillations using individualized strategies. We had Osc+ participants try five different breathing cycles from 9 to 13 s per breath and selected the pace that produced the largest amplitude oscillations at the breathing frequency (as indicated by spectral power at around that frequency), suggesting resonance between the baroreflex and breathing. For instance, if an Osc+ participant’s resonance frequency appeared to be 10 s (or 0.1 Hz) on the basis of high heart rate oscillations when breathing at that frequency, the participant was guided to inhale for 5 s and exhale for 5 s during their home practice sessions. For Osc- participants, we had them try out a set of self-generated strategies to reduce heart rate oscillations. Their proposed strategies included imagining natural scenes, listening to calming sounds, and closing eyes. Among the strategies, we selected the one whose frequency power was spread over the broad range of frequencies without a dominant frequency peak. Participants in both groups received feedback using performance scores which were calculated to reflect the opposite goals of the two conditions.

**Figure 2:**
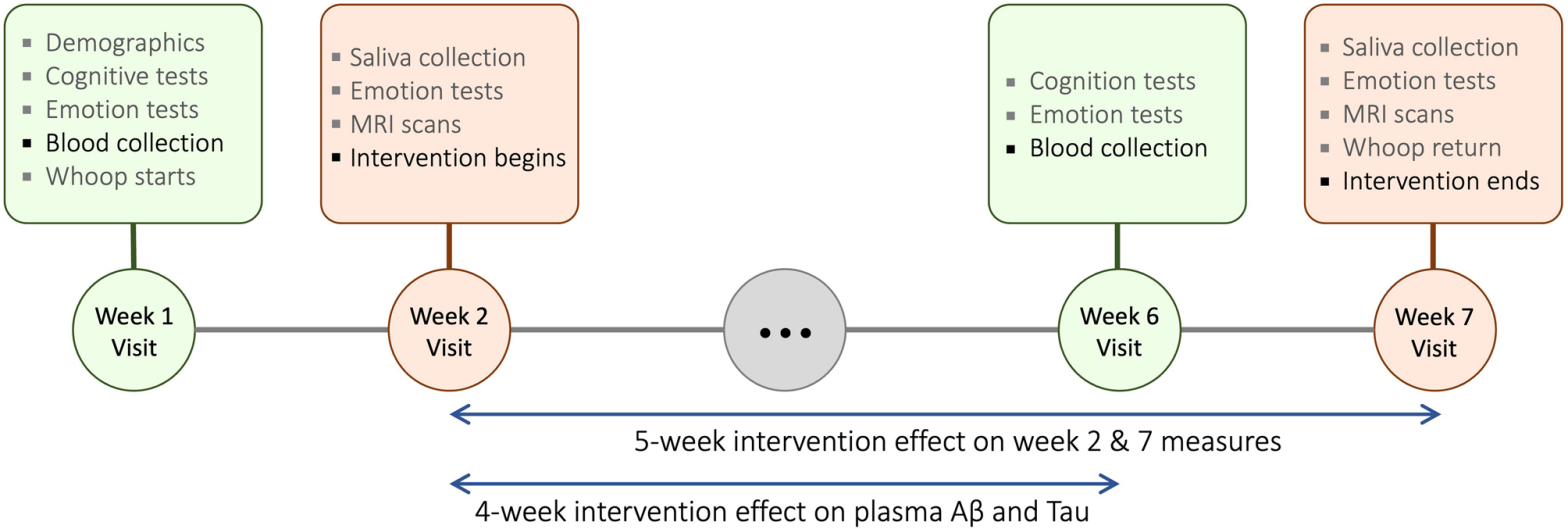
Weekly lab visit schedule. Schedules from Week 3 to 5 were not included because the visits were irrelevant to the measures reported in the current study. Detailed descriptions for each week can be found in the main outcome report of the intervention^30^.

**Figure 3:**
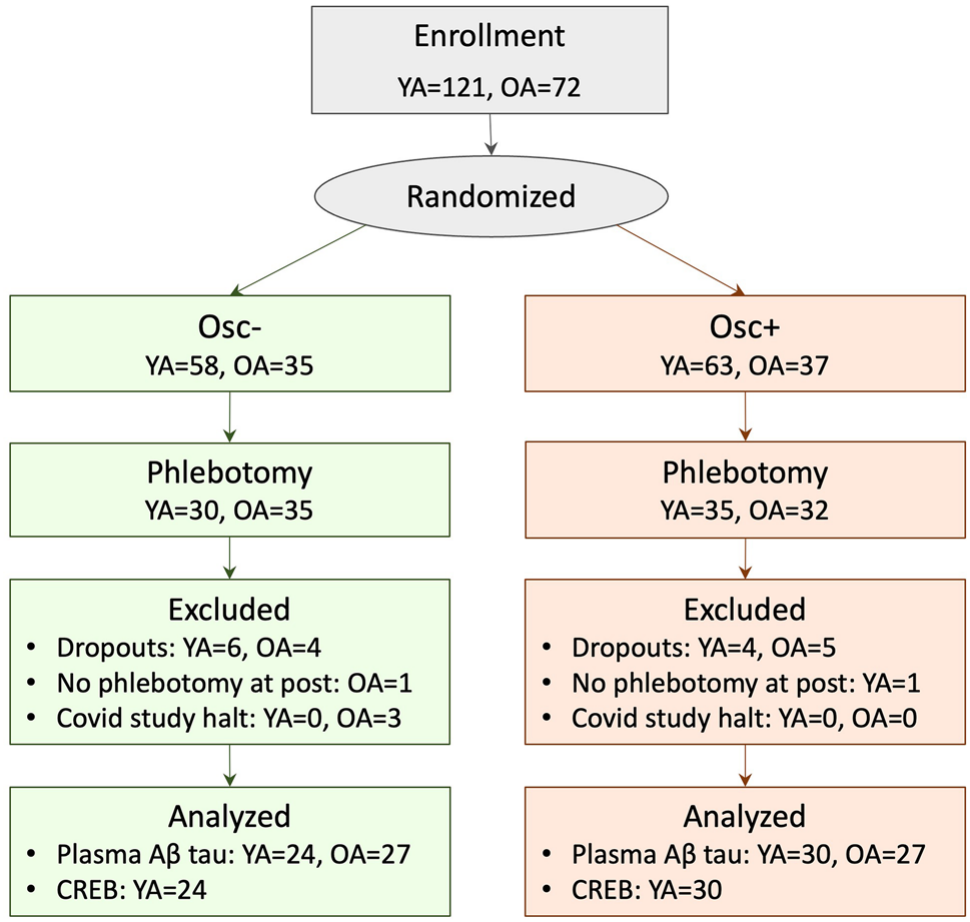
Flow chart. The phlebotomy took place for a subset of the total participants. The whole intervention had 15 dropouts for younger adults (7 Osc+, 8 Osc−) and 16 dropouts including 6 Covid-induced study halts for older adults (9 Osc+, 7 Osc−). Among 6 Covid-halted cases (all older adults), three participants were included for plasma assays because they completed up to Week 6 sessions including phlebotomy.

The whole study consisted of seven weekly visits. We collected baseline measurements during Week 1 and 2 visits and post-intervention measures during Week 6 and 7 visits. After Week 2 baseline measurements, participants were introduced to biofeedback training and took home a laptop computer connected with an ear sensor which measured their heartbeats and displayed real-time heart rate biofeedback on the screen. Participants were asked to practice their assigned intervention technique at home for at least 20 min every day from Week 2 until Week 7. The whole intervention lasted for 5 weeks from Week 2 through Week 7. However, we collected blood samples in Weeks 1 and 6. Since the intervention began on Week 2 and post-intervention blood draw took place on Week 6, the intervention effects on plasma Aβ and tau levels are based on 4 weeks of practice (Fig. 2). Upon completion of the study, participants were paid for their time and performance. The sample size for the intervention study was determined to detect medium effect size differences between the two groups. While we aimed for 100 younger and 100 older adults, a total of 106 younger and 56 older adults completed the whole HRV biofeedback intervention sessions lasting from Week 1 through Week 7. The number of younger participants from whom we collected blood samples was half of those who completed the whole study, because the blood collection setup was implemented halfway through the collecting of younger adult data. Due to Covid19, data collection for older adults was terminated before reaching the goal of 100. This yielded a final sample of 54 older and 54 younger participants available for plasma assays at both pre- and post-intervention (Fig. 3).

### Blood collection procedure

The blood samples were collected via antecubital venipuncture during Week 1 and 6 visits. A phlebotomist drew 10 ml of blood from each participant’s arm into a K2 EDTA tube and then 2.5 ml of blood into a PAXgene RNA tube. To separate plasma from red blood cells, the whole blood from the K2 EDTA tubes was centrifuged at the speed of 1500 RPM for 15 min at room temperature (15 °C). Plasma was then aliquoted in cryovials and stored at −80 °C. The frozen plasma samples were transferred and assayed at University of California, Irvine for Aβ42, Aβ40, tTau, and pTau-181. The PAXgene RNA tubes were gently inverted ten times right after sample collection and kept at room temperature for between 2.0 and 70.2 h (mean = 6.95 h) to comply with the PAXgene RNA tube requirement of 2- to 72-h stabilization time. The samples were then temporarily stored in a −20 °C freezer before being stored at −80 °C. They were transported for analysis to the Social Genomics Core Lab at University of California, Los Angeles.

### Plasma assay procedure

Plasma samples for both younger and older adults were analyzed using the automated Simoa SR-X analyzer with the commercially available Simoa Human Neurology 3-Plex A assay kit (Quanterix, Billerica, MA, USA) for Aβ42, Aβ40, and tTau. Plasma concentrations of pTau-181 were measured using the automated Simoa HD-X analyzer and the Simoa pTau-181 Advantage V2 kit (Quanterix, Billerica, MA, USA). Analyses were performed in duplicates (mean % coefficient of variation [%CV]: Aβ42: 6.33, Aβ40: 4.55, tTau: 8.81, pTau-181: 6.01) using a 1:4 dilution protocol according to the manufacturer's instructions. Prior to analysis, plasma samples were thawed at room temperature for 1 h and centrifuged at 10,000×*g* for 5 min to prevent transfer of debris. All assays were conducted by the same operator with the same respective instrument.

### PAXgene RNA assay procedure

The PAXgene RNA tubes of younger adults were transferred and assayed in a single batch using previously published methods^37^ which first extracted total RNA from samples using automated nucleic acid processing (QIAcube; Qiagen) and checked for suitable RNA integrity and mass (> 50 ng by NanoDrop One spectrophotometry; achieved mean = 4497 ng). Samples were then assayed by RNA sequencing using Lexogen QuantSeq 3’ FWD cDNA library synthesis and multiplex DNA sequencing on an Illumina HiSeq 4000 instrument with single-strand 65-nt sequence reads (all following the manufacturer’s standard protocol). Analyses targeted > 10 million sequence reads per sample (achieved mean 15.1 million), each of which was mapped to the RefSeq human transcriptome sequence using the STAR aligner (achieved average 94% mapping rate) to generate transcript counts per million total transcripts (TPM). TPM values were floored at 1 TPM to reduce spurious variability, log2-transformed to reduce heteroscedasticity, and analyzed by linear statistical models.

### Negative affect measure

During weekly lab visits for the pre- and post-intervention measures, we asked participants to take affect-related questionnaires including State Trait Anxiety Inventory (STAI)^64^, Center for Epidemiological Studies-Depression (CESD)^65^, Profile of Mood State (POMS)^66^. As POMS includes multiple mood states, we only averaged negative mood items for this analysis. As the three types of questionnaires were measured at Week 1 and 2, we had a total of six pre-intervention emotion measures for each participant. Likewise, we also have a total of six post-intervention emotion measures. To create summary scores for negative affect, we first standardized each measurement separately for younger and older participants. We next ran a principal component analysis on the six normalized pre-intervention measures separately for younger and older adults. This resulted in the pre-intervention summary score as well as one dominant component whose coefficient matrix elements indicate factor loadings for the six emotional measures. Using those matrix elements as weights, we calculated the weighted average of the six corresponding normalized post-intervention emotional measures (Week 6 and 7) to obtain post-intervention summary scores.

### Other physiological measures

Participants wore a photoplethysmography WHOOP wristband^67^ for the whole study period (Week 1 through Week 7) to assess changes in sleep hours and sleep-derived HRV. WHOOP algorithms have been validated as having a 95% sensitivity for sleep, 68% sensitivity for deep sleep and 70% for REM sleep^68^. Participants were instructed to wear the waterproof wristband as much as possible and especially during sleep.

Saliva samples for cortisol awakening response were collected only for younger adults to reduce participant burden for older adults. We instructed younger participants to collect saliva samples using oral swabs both upon awakening and 30 min later. Saliva was collected in the morning on Week 2 and 7 and brought to the lab in a thermos with ice packs. Samples were stored at −20 °C until they were sent to Salimetrics (CA, USA) for cortisol assays.

During Weeks 2 and 7 visits, we obtained resting heart rate data by having participants sit in a chair for five minutes. Using HeartMath emWave Pro software and its infrared pulse plethysmograph (PPG) ear sensor, the heartbeat was sampled at 370 Hz and its inter-beat interval data was recorded after removing artifacts. The data for each participant was analyzed with Kubios HRV Premium Version 3.1 to obtain a mean heart rate and root mean squared successive difference.

### Statistical analysis

All statistical analyses were performed using IBM SPSS Statistics (Version 28). To test the intervention effect, we ran a 2 (condition: Osc+, Osc−) × 2 (timepoint: pre-, post-intervention) ANOVA to test the intervention effect on each AD biomarker and followed each of these up with another 2 (condition: Osc+, Osc−) × 2 (timepoint: pre-, post-intervention) ANOVA for each age group (older and younger participants). We also tested whether our results still held when outliers were removed (Supplementary Information). Based on the boxplot rule of SPSS, outliers for each biomarker were identified if either their pre-intervention or post-intervention values among 108 participants were greater than the third quartile plus three times of interquartile range and lower than the first quartile minus three times of the interquartile range. We found two outliers for Aβ42 (one older Osc+, one older Osc−), one outlier for Aβ40 (one older Osc−), one outlier for Aβ42/40 ratio (one younger Osc−), six outliers for tTau (one younger Osc+, two younger Osc−’s, one older Osc+, two older Osc−’s), three outliers for pTau (two older Osc+’s, one older Osc−), and two outliers for pTau/tTau ratio (one younger Osc+, one older Osc+).

To compare the baseline characteristics of participants between the Osc+ and Osc− conditions, we ran independent sample *t*-tests on measures regarding heart rate, body mass index, and sleep with two-tailed *p* values (Table 2). We also compared males versus females on the same measures at baseline (Supplementary Information). We calculated change values by subtracting baseline values from the post-intervention values of the same measures in Table 2. Using these change values, we repeated two-tailed independent sample *t*-tests to compare Osc+ and Osc− (Table 3). We found that two older adults had abnormally extreme RMSSD values (> 270) at baseline and excluded them for the heart rate and RMSSD comparisons in older adults in Tables 2 and 3.

We examined how changes in each of the six plasma biomarkers (Aβ42, Aβ40, Aβ42/Aβ40 ratio, tTau, pTau-181, and pTau/tTau ratio) related to changes in CREB-regulated gene transcription activity in an exploratory analysis with the younger adults. The analysis employed the Transcription Element Listening System (TELiS)^69,70^ approach to identify transcription-factor binding motifs (TFBMs) that were overrepresented among the promoters of up- or down-regulated genes in association with each of the six plasma biomarker change scores (post–pre intervention). We examined the relationship between change in plasma biomarkers and change in CREB-regulated gene transcription activity that mediates sympathetic nervous system-induced β-adrenergic signaling, with statistical significance evaluated using standard errors derived by bootstrapping (200 cycles).

To test whether the intervention changed the levels of negative affect, we ran a 2 (condition: Osc+, Osc−) × 2 (timepoint: pre-, post-intervention) ANOVA with negative affect summary scores as the dependent measure separately for younger and older adults. To explore how individual differences in change of negative affect are associated with individual differences in change of Aβ42/40 ratio, we ran a partial correlation between the post-intervention negative affect summary score and post-intervention Aβ42/40 ratio separately for younger and older adults while controlling for the pre-intervention Aβ42/40 and negative affect values.

### Corrections for multiple comparisons

We used the false discovery rate method to correct for multiple comparisons in our study^44^. The false discovery rate is the expected fraction of tests from an analysis set that are declared significant for which the null hypothesis is true. The major benefit of the false discovery rate correction is that it allows for appropriately different thresholds for: (1) a set of analyses with scientifically driven hypotheses for which the null hypotheses are generally false vs. (2) a set of exploratory analyses for which null hypotheses are generally true. The false discovery rate sets the thresholds based on the nature of the distributions of *p* values from the set of tests^71^.

We controlled for false discovery rate at the 0.05 level for our 36 hypothesis-driven tests. These consisted of (1) 19 tests of a time-point by condition interaction: six biomarkers (Aβ42, Aβ40, Aβ42/Aβ40 ratio, tTau, pTau-181, and pTau/tTau ratio) × 3 tests (for all, younger, and older participants) and CREB (available only for younger participants), and (2) 17 tests of association: six CREB and biomarker change associations, three correlations between changes in the pTau/Tau ratio and Aβ42/Aβ40 ratio (for all, younger and older participants), six age-group differences in biomarkers at baseline, and two correlations between negative affect change and Aβ42/Aβ40 ratio change (for younger and older participants). For these 36 tests which are not independent from each other, we report adjusted *p* values obtained from the two-stage Benjamini and Hochberg linear step-up procedure (‘TSBH’)^44^ using the R package, ‘multtest’ (Version 2.54.0)^43,72^.

We note that we also included a series of non-hypothesis-driven tests listed in Tables 2 and 3. Table 2 consists of the baseline comparisons of various measures across the two conditions separately by age group. For the baseline tests, the conservative thing to do is not to correct for multiple comparisons, as they serve as control variables for us to detect whether there were potential problems with randomization. Thus, for Table 2, we did not employ false discovery rate control. Table 3 compares pre vs. post changes across conditions for 26 measures for which we had no specific hypotheses. For the Table 3 set of tests, we did not correct for multiple comparisons to increase the chance of detecting potentially confounding factors. However, none of the tests turned out significant before corrections. For all these tests in Tables 2 and 3, we report original *p* values.

### Ethical approval and consent to participate

The study was approved by the institutional Review Board at University of Southern California (ID: UP-17-00219, Name: HRV-biofeedback and emotion regulation). We obtained written informed consent forms from all participants of the study. All the experiments involving human blood samples were carried out in accordance with relevant guidelines and regulations. The experimental procedures were specified in the Biohazardous Use Application (BUA-18-00027), approved by the Institutional Biosafety Committee at University of Southern California.

## Supplementary Information

Supplementary Information.

## Data Availability

Upon publication, study data (i.e., individual participant assay values) will be made publicly available at OpenNeuro as part of the parent study dataset: https://openneuro.org/datasets/ds003823.

## Abbreviations

Aβ: Amyloid-beta
AD: Alzheimer’s disease
ANOVA: Analysis of variance
CESD: Center for Epidemiological Studies-Depression
CREB: CAMP response element-binding protein
CSF: Cerebrospinal fluid
HRV: Heart rate variability
Osc−: The current control condition for decreasing heart rate oscillations
Osc+: The current intervention condition for increasing heart rate oscillations
PET: Positron emission tomography
POMS: Profile of Mood State
pTau: Phosphorylated tau
RNA: Ribonucleic acid
STAI: State Trait Anxiety Inventory
TELiS: Transcription Element Listening System
TFBMs: Transcription-factor binding motifs
TPM: Transcript counts per million total transcripts
tTau: Total tau
