# Supplementary material for: Modulating heart rate oscillation affects plasma amyloid beta and tau levels in younger and older adults
Source: Sci Rep. 2023 Mar 9;13:3967. doi: 10.1038/s41598-023-30167-0 (PMC9998394; doi:10.1038/s41598-023-30167-0)
Supplement: Supplementary file 1 — Supplementary Information. [file 41598_2023_30167_MOESM1_ESM.docx]

**SUPPLEMENTARY INFORMATION**

**Results after removing outliers for Aβ and tau**

We examined whether removing the extreme outliers influence the significance of the interaction effect in the plasma Aβ and tau levels. When we excluded them (2 for Aβ42, 1 for Aβ40), the significant time-point X condition interactions held up for Aβ42, *F*(1, 104) = 22.23, *p* < 0.001, *η_p_*^2^ = 0.18, and for Aβ40, *F*(1, 105) = 16.56, *p* < 0.001, *η*_p_^2^ = 0.14. When we excluded extreme outliers in tau levels (6 for tTau, 3 for pTau-181), the time-point X condition interactions stayed similar for tTau, *F*(1, 100) = 4.77, *p* = 0.03, *η*_p_^2^ = 0.05, and for pTau-181, *F*(1, 103) = 1.04, *p* = 0.31, *η*_p_^2^ = 0.01. Regarding the Aβ42/Aβ40 and pTau-181/tTau ratios, removing the outliers (1 for Aβ42/Aβ40, 2 for pTau-181/tTau) did not change the significance of the relationship, *F*(1, 105) = 0.57, *p* = 0.45, *η*_p_^2^ = 0.005 for Aβ42/Aβ40 and *F*(1, 104) = 10.38, *p* = 0.002, *η*_p_^2^ = 0.09 for pTau-181/tTau.

In the follow-up analyses splitting participants into two age groups, we also examined whether the outliers influenced the significance of the interaction. Removing the outliers (2 older adults) did not change the significance for Aβ42, *F*(1, 52) = 12.30, *p* < 0.001, *η*_p_^2^ = 0.19 for younger adults and *F*(1, 50) = 10.67, *p* = 0.002, *η*_p_^2^ = 0.18 for older adults. Removing the outliers (1 older adult) did not change the significance in Aβ40, *F*(1, 52) = 7.37, *p* = 0.009, *η*_p_^2^ = 0.12 for younger adults and *F*(1, 51) = 10.36, *p* = 0.002, *η*_p_^2^ = 0.17 for older adults. The significance level in Aβ42/Aβ40 ratio was not influenced by removing the outliers (1 younger adult), *F*(1, 51) = 0.013, *p* = 0.91, *η*_p_^2^ < 0.001 for younger adults and *F*(1, 52) = 3.35, *p* = 0.07, *η*_p_^2^ = 0.06 for older adults. After removing the outliers (3 younger adults and 3 older adults) in the tTau levels, the significance did not change for younger adults, *F*(1, 49) = 7.93, *p* = 0.007, *η*_p_^2^ = 0.14, and older adults, *F*(1, 49) = 0.07, *p* = 0.80, *η*_p_^2^ = 0.001. In the pTau-181 levels, removing the outliers (3 older adults) changed the results for older adults, *F*(1, 49) = 2.37, *p* = 0.13, *η*_p_^2^ = 0.05, and no changes for younger adults, *F*(1, 52) = 0.02, *p* = 0.88, *η*_p_^2^ < 0.001. In the pTau/tTau ratio, removing the outliers (1 younger adult and 1 older adult) changed the significance in older adults, *F*(1, 51) = 6.71, *p* = 0.01, *η*_p_^2^ = 0.12, but did not in younger adults, *F*(1, 51) = 3.38, *p* = 0.07, *η*_p_^2^ = 0.06.

**Changes in Aβ and tau, resting-state HRV, and heart rate oscillations during practice**

We tested whether the changes in Aβ and tau are related with changes in resting-state heart rate and RMSSD by taking partial correlations between each pair’s post-intervention values when controlling for their pre-intervention values. We further examined whether changes in individual Αβ and tau levels are correlated with the average power around the breathing frequency range (log) by running correlations between the log average power and each biomarker’s post-intervention values when controlling for its pre-intervention values.

Regarding individual Aβ and tau changes, we found no correlations with resting-state physiological changes. Changes in mean heart rate were not correlated with changes in Aβ42, *r*(101) = -0.01, *p* = 0.90, Aβ40, *r*(101) = 0.07, *p* = 0.50, tTau, *r*(101) = 0.15, *p* = 0.14, and pTau-181, *r*(101) = -0.13, *p* = 0.20. No correlations were found between changes in RMSSD and changes in any plasma biomarkers including Aβ42, *r*(101) = 0.11, *p* = 0.27, Aβ40, *r*(101) = 0.05, *p* = 0.61, tTau, *r*(101) = -0.06, *p* = 0.56, pTau-181, *r*(101) = -0.01, *p* = 0.89. However, except for pTau-181, *r*(105) = -0.04, *p* = 0.70, we found that decreases in Αβ42, Αβ40, and tTau were correlated with the log average power during practice, *r*(105) = -0.23, *p* = 0.02 for Αβ42, *r*(105) = -0.28, *p* = 0.003 for Αβ40, and *r*(105) = -0.20, *p* = 0.04 for tTau, which suggests that plasma Αβ and tTau decreased as the heart rate oscillations during practice were more intense.

Interestingly, observations were reversed for the Aβ42/Aβ40 and pTau-181/tTau ratios. Changes in mean heart rate were correlated with changes in the Aβ42/Aβ40 ratio, *r*(101) = -0.26, *p* = 0.01, and changes in the pTau-181/tTau ratio, *r*(101) = -0.20, *p* = 0.05. No correlations were found between changes in RMSSD and changes in the Aβ42/Aβ40 ratio, *r*(101) = 0.11, *p* = 0.27, and the pTau/tTau ratio, *r*(101) = 0.08, *p* = 0.42. The log average power was not correlated with either the Αβ42/Αβ40, *r*(105) = 0.12, *p* = 0.20, nor with pTau-181/tTau ratios, *r*(105) = 0.09, *p* = 0.34.

**Supplementary Table. Sex differences at baseline**

| Younger Adults | Males | | Females | | Test statistics | | |
| --- | --- | --- | --- | --- | --- | --- | --- |
|  | M | SE | M | SE | *t* | *df* | *p* |
| Age (yr) | 22.76 | 0.63 | 22.59 | 0.54 | 0.19 | 52 | 0.85 |
| Plasma Ab42 (pg/ml) | 9.46 | 0.56 | 9.58 | 0.38 | -0.18 | 52 | 0.86 |
| Plasma Ab40 (pg/ml) | 150.64 | 11.00 | 164.98 | 4.64 | -1.20 | 23.2 | 0.12 |
| Plasma Ab42/Ab40 ratio | 0.06 | 0.002 | 0.06 | 0.003 | 1.65 | 52 | 0.11 |
| Plasma tTau (pg/ml) | 2.39 | 0.24 | 1.99 | 0.10 | 1.54 | 22.8 | 0.14 |
| Plasma pTau-181 (pg/ml) | 2.09 | 0.17 | 1.31 | 0.07 | 4.19 | 23.4 | < 0.001 |
| Plasma pTau/tTau ratio | 0.94 | 0.08 | 0.69 | 0.05 | 2.85 | 52 | 0.01 |
| Body mass index (kg/m^2^) | 24.43 | 0.82 | 22.93 | 0.76 | 1.25 | 51 | 0.22 |
| Waist hip ratio | 0.84 | 0.01 | 0.77 | 0.01 | 5.05 | 52 | < 0.001 |
| Systolic blood pressure (mmHg) | 120.75 | 2.14 | 108.21 | 1.48 | 4.85 | 52 | < 0.001 |
| Diastolic blood pressure (mmHg) | 74.58 | 1.60 | 67.71 | 1.10 | 3.57 | 52 | < 0.001 |
| Mean heart rate (beat/min) | 70.36 | 2.10 | 73.50 | 1.83 | -1.05 | 52 | 0.30 |
| RMSSD (ms) | 61.95 | 6.30 | 63.50 | 4.74 | -0.19 | 46.6 | 0.85 |
| Hours of sleep (hr) | 5.76 | 0.17 | 6.51 | 0.16 | -2.89 | 45 | 0.01 |
| Hours of REM sleep (hr) | 1.26 | 0.08 | 1.50 | 0.10 | -1.90 | 43.4 | 0.06 |
| Hours of deep sleep (hr) | 0.96 | 0.09 | 1.24 | 0.09 | -1.92 | 45 | 0.06 |
| Heart rate during deep sleep (beat/min) | 58.92 | 1.02 | 61.06 | 1.34 | -1.27 | 44.8 | 0.21 |
| RMSSD during deep sleep (ms) | 84.83 | 9.09 | 76.66 | 5.03 | 0.86 | 45 | 0.40 |
| Cortisol at awakening (µg/dl) | 0.22 | 0.04 | 0.30 | 0.03 | -1.63 | 49 | 0.11 |
| Cortisol awakening response (µg/dl) | 0.17 | 0.05 | 0.24 | 0.05 | -0.83 | 47 | 0.41 |
| Blood collection time (hr) | 14.49 | 0.16 | 14.32 | 0.10 | 0.89 | 52 | 0.38 |
|  |  |  |  |  |  |  |  |
| Older Adults | Males | | Females | | Test statistics | | |
|  | M | SE | M | SE | *t* | *df* | *p* |
| Age (yr) | 64.08 | 1.68 | 66.63 | 1.15 | -1.20 | 52 | 0.24 |
| Plasma Ab42 (pg/ml) | 10.78 | 0.99 | 10.89 | 0.45 | -0.12 | 52 | 0.90 |
| Plasma Ab40 (pg/ml) | 226.25 | 40.50 | 296.68 | 7.97 | 0.72 | 15.1 | 0.49 |
| Plasma Ab42/Ab40 ratio | 0.05 | 0.002 | 0.06 | 0.001 | -1.74 | 52 | 0.09 |
| Plasma tTau (pg/ml) | 2.14 | 0.30 | 2.00 | 0.13 | 0.51 | 52 | 0.61 |
| Plasma pTau-181 (pg/ml) | 3.02 | 0.47 | 2.43 | 0.18 | 1.43 | 52 | 0.16 |
| Plasma pTau/tTau ratio | 1.54 | 0.16 | 1.36 | 0.13 | 0.78 | 52 | 0.44 |
| Body mass index (kg/m^2^) | 29.13 | 1.73 | 25.45 | 0.91 | 2.04 | 52 | 0.05 |
| Waist hip ratio | 0.95 | 0.02 | 0.83 | 0.03 | 2.75 | 52 | 0.01 |
| Systolic blood pressure (mmHg) | 132.10 | 3.54 | 126.19 | 2.47 | 1.30 | 52 | 0.20 |
| Diastolic blood pressure (mmHg) | 82.70 | 2.41 | 75.99 | 1.30 | 2.61 | 52 | 0.18 |
| Mean heart rate (beat/min) | 68.51 | 3.43 | 70.74 | 1.49 | -0.70 | 47 | 0.48 |
| RMSSD (ms) | 32.48 | 3.14 | 34.80 | 3.17 | -0.43 | 47 | 0.67 |
| Hours of sleep (hr) | 5.85 | 0.47 | 5.83 | 0.18 | 0.05 | 43 | 0.96 |
| Hours of REM sleep (hr) | 1.46 | 0.15 | 1.50 | 0.09 | -0.20 | 43 | 0.84 |
| Hours of deep sleep (hr) | 1.20 | 0.10 | 1.13 | 0.05 | 0.66 | 43 | 0.52 |
| Heart rate during deep sleep (beat/min) | 60.55 | 3.34 | 61.99 | 1.22 | -0.51 | 43 | 0.61 |
| RMSSD during deep sleep (ms) | 65.56 | 9.13 | 46.99 | 4.68 | 1.96 | 43 | 0.06 |
| Blood collection time (hr) | 12.30 | 0.34 | 12.00 | 0.18 | 0.84 | 52 | 0.41 |
